# Increased Levels of Antigen-Bound β-Amyloid Autoantibodies in Serum and Cerebrospinal Fluid of Alzheimer’s Disease Patients

**DOI:** 10.1371/journal.pone.0068996

**Published:** 2013-07-18

**Authors:** Madalina Maftei, Franka Thurm, Cathrin Schnack, Hayrettin Tumani, Markus Otto, Thomas Elbert, Iris-Tatjana Kolassa, Michael Przybylski, Marilena Manea, Christine A. F. von Arnim

**Affiliations:** 1 Laboratory of Analytical Chemistry and Biopolymer Structure Analysis, Department of Chemistry, University of Konstanz, Konstanz, Germany; 2 Steinbeis Research Center for Biopolymer Analysis, University of Konstanz, Konstanz, Germany; 3 Department of Psychology, University of Konstanz, Konstanz, Germany; 4 Clinical and Biological Psychology, Institute of Psychology and Education, University of Ulm, Ulm, Germany; 5 Department of Psychology, TU Dresden, Dresden, Germany; 6 Department of Neurology, University of Ulm, Ulm, Germany; 7 Zukunftskolleg, University of Konstanz, Konstanz, Germany; Julius-Maximilians-Universität Würzburg, Germany

## Abstract

Recent studies have suggested a protective role of physiological β-amyloid autoantibodies (Aβ-autoantibodies) in Alzheimer’s disease (AD). However, the determination of both free and dissociated Aβ-autoantibodies in serum hitherto has yielded inconsistent results regarding their function and possible biomarker value. Here we report the application of a new sandwich enzyme-linked immunosorbent assay (ELISA) for the determination of antigen-bound Aβ-autoantibodies (intact Aβ-IgG immune complexes) in serum and cerebrospinal fluid (CSF) of a total number of 112 AD patients and age- and gender-matched control subjects. Both serum and CSF levels of Aβ-IgG immune complexes were found to be significantly higher in AD patients compared to control subjects. Moreover, the levels of Aβ-IgG complexes were negatively correlated with the cognitive status across the groups, increasing with declining cognitive test performance of the subjects. Our results suggest a contribution of IgG-type autoantibodies to Aβ clearance *in vivo* and an increased immune response in AD, which may be associated with deficient Aβ-IgG removal. These findings may contribute to elucidating the role of Aβ-autoantibodies in AD pathophysiology and their potential application in AD diagnosis.

## Introduction

Alzheimer’s disease (AD) is the most prevalent form of dementia among the aging population. Its long preclinical phase and the lack of biomarkers that would allow an early diagnosis pose great challenges for the development of effective therapeutic approaches. The neuropathology of AD is characterized by the accumulation of intracellular neurofibrillary tangles and extracellular beta-amyloid (Aβ) plaques, associated with axonal, dendritic and synaptic degeneration [1]–[4]. Several species of aggregated Aβ, such as small oligomers, annular oligomers and fibrils, precede the formation of amyloid plaques in the AD brain. The small Aβ oligomers, consisting of 3–50 monomer units, appear to be the most neurotoxic species [5].

In transgenic mouse models of AD, both active immunization with full-length Aβ peptides or Aβ fragments [6]–[9] and passive immunization with monoclonal anti-Aβ-antibodies [10]–[12] were effective in preventing Aβ-aggregation, clearing amyloid plaques and improving cognitive performance. Based on the promising preclinical results, immunotherapy has been proposed as a possible therapeutic approach for AD [13], [14]. A phase II multicenter clinical trial of active immunization with preaggregated Aβ42 (AN1792(QS-21) vaccine) showed a reduction of amyloid plaque burden and slower cognitive decline in AD patients. However, the trial was interrupted due to the occurrence of meningoencephalitis in some of the immunized participants [14], [15]. A follow-up study of the AN1792 clinical trial with yearly assessments and post-mortem neuropathological examinations indicated progression of AD-related neurodegeneration and cognitive decline, despite vaccination [16]. Another study reporting the clinical effects of a phase IIa immunotherapeutic trial of AN1792 showed similar results, but also revealed a significantly higher score in one of the neuropsychological test batteries in antibody responders compared to the placebo group, suggesting that Aβ-immunotherapy may be useful for the treatment of AD [17]. Several clinical trials are carried out to further evaluate the therapeutic potential of Aβ-based active immunization and to assess the effect of passive immunization with anti-Aβ-antibodies in AD patients [18]. Two phase 3 clinical trials designed to evaluate the efficacy and safety of a humanized N-terminal anti-Aβ monoclonal antibody, Bapineuzumab, in patients with mild to moderate AD have recently been completed (http://clinicaltrials.gov). Results presented at the 16^th^ EFNS congress in Stockholm showed that the treatment with Bapineuzumab did not reach clinical endpoints (no significant benefit on cognitive or functional performance); however, reduced CSF levels of phospho-tau were observed in the Bapineuzumab-treated group (http://www.stevenderoover.be/EFNS/Presentations/EFNS2012/WC220/; http://www.stevenderoover.be/EFNS/Presentations/EFNS2012/WC219/). Considering the difficulty to find an efficient treatment that would improve the cognitive functions of AD patients, a promising approach would be the administration of potential drugs (e.g., antibodies) at the earliest possible stage, before or just after the onset of AD symptoms, in order to prevent the disease progression [19].

Recently, physiological antibodies binding Aβ (Aβ-autoantibodies) have been detected in serum and CSF of AD patients and healthy individuals [20]–[24], as well as in intravenous immunoglobulin preparations (IVIg), which are fractionated blood products used for the treatment of immune deficiencies and other disorders [25]. Dodel et al. [26] reported that administration of Aβ-autoantibodies led to reduced plaque formation and improvement of behavior in a mouse model of AD. Moreover, in AD patients, promising effects on cognition were observed in small pilot trials involving passive immunization with IVIg [20], [23], [27]. These findings suggest that Aβ-autoantibodies might exert a protective function against AD and could play an important role in AD treatment.

In addition to their potential therapeutic applications for AD, the biomarker value of Aβ-autoantibodies was also investigated. Currently available data on the serum/plasma levels of Aβ-autoantibodies in AD patients compared to healthy individuals are controversial. Several groups found that the serum levels of free, non-antigen-bound Aβ-autoantibodies were lower in AD patients than in controls [28]–[30], while others reported either higher values [31] or no difference [32], [33]. So far, there is only one reported study on the CSF levels of free Aβ-autoantibodies, showing decreased values in AD patients compared to control subjects [34]. Gustaw et al. [35] suggested that the presence of Aβ-autoantibodies not only in free, non-antigen-bound state, but also as preformed immune complexes with Aβ-peptides, may be a possible reason for these controversial results. Subsequent serum determinations of Aβ-autoantibodies after acidic dissociation of the Aβ-immune complexes indicated higher levels of Aβ-autoantibodies in AD patients compared to controls [35], [36]. However, using a similar procedure, Klaver et al. [37] found no difference between the groups.

Based on the finding that Aβ-autoantibodies recognize the Aβ (21–37) epitope [38], [39], unlike the antibodies produced by active immunization that bind the Aβ (4–10) epitope [40], we have recently developed a sandwich ELISA for the determination of intact Aβ-IgG immune complexes and applied it for the analysis of serum samples from healthy individuals aged 18 to 89 years. The serum levels of Aβ-IgG immune complexes were not correlated with age or cognitive performance of healthy adults [41]. To date, there are no other reports on the determination of Aβ-IgG immune complexes in serum or CSF by ELISA.

In the present study, we have employed the sandwich ELISA to determine the levels of Aβ-IgG immune complexes in both serum and CSF of AD patients and age- and gender-matched control subjects and evaluated their correlations with the neuropsychological performance and age of the study participants, as well as their diagnostic power.

## Materials and Methods

### Ethics Statement

This study was approved by the ethics committee of the University of Ulm, Germany, and conducted according to the guidelines outlined in the Declaration of Helsinki. Prior to participation, written informed consent was obtained.

### Participants

Demographic data are depicted in Table 1. Altogether, 58 AD patients were recruited at the Memory Clinic of the Hospital for Neurology of the University of Ulm, Germany. Patients underwent a comprehensive clinical neurological examination, a routine blood analysis, structural imaging (MRI or CT), apolipoprotein E (APOE) genotyping and a detailed neuropsychological assessment, including the Mini Mental State Examination test (MMSE, range 0–30 points; [42]) and the Alzheimer’s Disease Assessment Scale - Cognitive subscale (ADAS-Cog, range 0–70 errors; e.g., [43]). Probable AD was diagnosed according to NINCDS-ADRDA [44] and DSM-IV-TR criteria [45]. Furthermore, 54 unrelated age- and gender-matched control subjects were recruited at the same site and did not display any cognitive or neurological deficits following thorough clinical and neuropsychological examination.

**Table 1:** Demographic and clinical characteristics of Alzheimer’s disease patients (AD) and controls (C).

	Serum donors database		CSF donors database	
	AD (*n* = 45)	C (*n* = 42)	AD (*n* = 37)	C (*n* = 29)
Age (years)	70.0±7.5	68.7±7.4	69.3±7.4	71.1±5.8
Gender (% male)	33.3	33.3	40.5	51.7
APOE (% ε4)	58.5	15.2	52.9	16.7
	(*n* = 41)	(*n* = 33)	(*n* = 34)	(*n* = 12)
MMSE	19.7±4.4	29.2±0.8	19.6±5.2	28.8±1.4
	(*n* = 44)	(*n* = 37)	(*n* = 37)	(*n* = 24)
ADAS-Cog	27.0±8.3	8.8±2.9	24.7±10.8	8.5±3.6
	(*n* = 30)	(*n* = 25)	(*n* = 20)	(*n* = 11)
CSF Aβ42 (pg/mL)	499±177	999±322	520±193	951±327
	(*n* = 44)	(*n* = 36)	(*n* = 36)	(*n* = 23)
CSF T-tau (pg/mL)	786±381	288±132	744±377	300±109
	(*n* = 44)	(*n* = 36)	(*n* = 36)	(*n* = 23)
Serum Aβ-IgG (OD)^a^	0.569±0.2	0.463±0.2		
	(*n* = 45)	(*n* = 42)		
CSF Aβ-IgG (OD)^b^			0.449±0.2	0.348±0.2
			(*n* = 37)	(*n* = 29)

Values are mean ± standard deviation. For gender and APOE status, percentages per group are given. ADAS-Cog - Alzheimer Disease Assessment Scale-Cognitive Subscale (range 0–70 errors); MMSE - Mini Mental Status Examination (range 0–30 points); OD - optical density.

a Diluted 1∶100.

b Diluted 1∶1.

### Determination of Aβ42 and Total tau (T-tau) Levels in CSF

The collection of CSF samples by lumbar puncture and the pre-analytical processing were performed using a standardized protocol [46]. In brief, CSF samples were collected into polypropylene tubes, centrifuged immediately and stored at −80°C. The CSF levels of total tau (T-tau) were determined using a sandwich ELISA (INNOTEST® hTau Ag, Innogenetics, Belgium), by which both normally phosphorylated and non-phosphorylated tau were detected. The assay was performed according to the manufacturers’ instructions and the laboratory reference ranges were as follows: <200 ng/L and <300 ng/L for control individuals below 65 and older than 65 years, respectively. The concentrations of total tau in the analyzed CSF samples were estimated from standard curves obtained for each assay. Tau levels >350 ng/L were regarded as indicative of a neurodegenerative process. The analytical sensitivity of the assay was 75 pg/mL, and the intra-assay and inter-assay variations were <8%. The CSF levels of Aβ (1–42) (Aβ42) were determined using a commercially available sandwich ELISA kit (INNOTEST® β-amyloid(1–42), Innogenetics, Belgium), according to the protocol supplied with the kit. CSF Aβ42 concentrations of the samples were estimated from standard curves obtained for each assay. Aβ42 levels below 550 ng/L were regarded as abnormal.

### ELISA Determination of Aβ-IgG Immune Complexes in Serum and CSF

**Figure 1:**
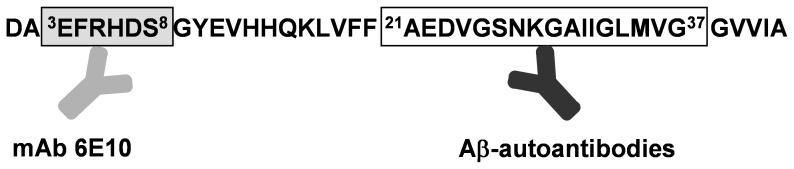
Epitope specificities of the coating antibody (mAb 6E10) and the Aβ-autoantibodies.

Serum levels of Aβ-IgG immune complexes were determined by sandwich ELISA, as previously reported [41]. The method is based on the different epitope specificities of the Aβ-autoantibodies, that recognize Aβ (21–37) and of a mouse monoclonal 6E10 antibody (mAb 6E10), that binds to Aβ (3–8) (Figure 1). Briefly, 96-well ELISA plates were coated overnight with the mAb 6E10 (Covance, Emeryville, CA, USA) followed by blocking the unspecific binding sites with 5% bovine serum albumin (BSA, w/v), 0.1% Tween-20 (v/v) in phosphate buffered saline (PBS, pH 7.4). Subsequently, human serum samples, diluted 1∶100 with blocking buffer, were applied in triplicates. For detection, a horseradish peroxidase (HRP)-conjugated goat anti-human IgG (H+L) antibody (Pierce, Rockford, IL, USA) showing no cross-reactivity with mouse IgG and *o*-phenylenediamine (OPD, Merck, Darmstadt, Germany) as enzymatic substrate were used. The optical density (OD) was measured at 450 nm on a Wallac 1420 Victor^2^ ELISA Plate Counter (Perkin Elmer, Rodgau, Germany). The described sandwich ELISA was also optimized for the analysis of CSF samples. Two washing buffers, PBS-Tween (0.05% Tween-20 in PBS, v/v) and PBS-Triton (0.1% Triton X-100 in PBS, v/v) and various CSF dilutions (1∶300, 1∶100, 1∶30, 1∶10, 1∶3 and 1∶1) were tested. The highest OD response was obtained using PBS-Tween for washing and 1∶1 CSF dilution.

For both serum and CSF determinations, triplicate 3-fold dilutions from a stock solution (7 µg/µL in blocking buffer) of human serum γ-globulin (immunoglobulin preparation, Calbiochem, Merck, Darmstadt, Germany) were used as reference, to allow data to be normalized between plates and different experiments. The non-specific binding (NSB) of the IgG preparation and analyte samples was assessed from triplicate wells containing all components except the mAb 6E10. The average OD values, NSB subtraction, standard deviation (SD) and intra−/inter-assay coefficients of variation (CV) were calculated with the WorkOut2.0 software (Perkin Elmer, Rodgau, Germany). Both serum and CSF determinations of Aβ-IgG immune complexes showed intra-assay CVs<10% and inter-assay CVs<15%. For the newly developed CSF ELISA, the cut-off values of the assay, defined as the linearity limits of the reference curve (R^2^>0.97), were as follows: 0.065 min. and 1.129 max. Since there is no unique method for expressing ELISA responses and arbitrary units are derived from absorbance readings, we considered it adequate to present the results of Aβ-IgG determinations in serum and CSF as OD values.

### Data Analysis

Statistical analysis was performed using the R statistical software package of The R Foundation of Statistical Computing (www.r-project.org; version 2.11.1 for Mac OS X, GUI 1.34 Leopard).

Welch’s two-sample *t*-tests (two-tailed with modified degrees of freedom) were applied to examine differences in demographic and cognitive data between AD patients and controls. Analysis of variance with group as factor and age as covariate were computed in order to investigate differences in the levels of Aβ-IgG immune complexes between both groups. Models’ residuals were tested for normality using the Shapiro-Wilk normality test. For categorical variables, Pearsons’s Chi-squared (*χ* ^2^) test was computed. Pearson's *r* product moment correlation coefficient was calculated in order to investigate possible associations of serum and CSF levels of Aβ-IgG immune complexes with age and neuropsychological performance (MMSE, ADAS-Cog). The diagnostic power of the Aβ-IgG immune complexes in serum and CSF was calculated using receiver-operating characteristic (ROC) curve analysis (package Daim and pROC for R; [47], [48]). All tests for statistical significance referred to a significance level with *α* ≤0.05.

## Results

### Demographic and Clinical Characteristics of Alzheimer’s Disease Patients and Controls

Demographic and clinical characteristics of Alzheimer’s disease patients and controls are shown in Table 1 and Table S1 in the Supporting Information. The statistical evaluation indicated a similar distribution of age (*t*_(85)_ = 0.79, *p* = 0.43 for serum donors; *t*_(64)_ = −1.12, *p* = 0.27 for CSF donors) and gender (*χ* ^2^_(1)_ = 0.05, *p* = 0.82 for serum donors; *χ* ^2^_(1)_ = 0.43, *p* = 0.51 for CSF donors) in the AD and the control group. As expected, AD patients scored lower in the MMSE (*t*_(47)_ = −14.00, *p*<0.0001 for serum donors; *t*_(44)_ = −10.16, *p*<0.0001 for CSF donors) and committed more errors in the ADAS-Cog neuropsychological test battery (*t*_(37)_ = 11.30, *p*<0.0001 for serum donors; *t*_(26)_ = 6.12, *p*<0.0001 for CSF donors) than the control subjects. They also presented significantly lower levels of Aβ42 (*t*_(69)_ = −9.39, *p*<0.0001) and higher levels of T-tau (*t*_(69)_ = 8.88, *p*<0.0001) in CSF. Furthermore, an increased incidence of APOE ε4 allele was observed in the AD cases (*χ* ^2^_(1)_ = 15.26, *p*<0.0001).

In the following paragraphs we compare the levels of Aβ-IgG immune complexes in serum and CSF samples from AD patients and age- and gender-matched control subjects. Since old age and APOE ε4 status are considered to be associated with an increased risk of AD pathology [49], we also included age as covariate into the group comparison and further investigated potential differences between the levels of Aβ-IgG immune complexes in serum and CSF with respect to APOE genotype.

### Aβ-IgG Immune Complexes in Serum of AD Patients and Control Subjects

Two samples (from one AD patient and one control subject) were excluded from the statistical analysis, since the Aβ-IgG levels exceeded the ELISA cut-off values.

**Figure 2:**
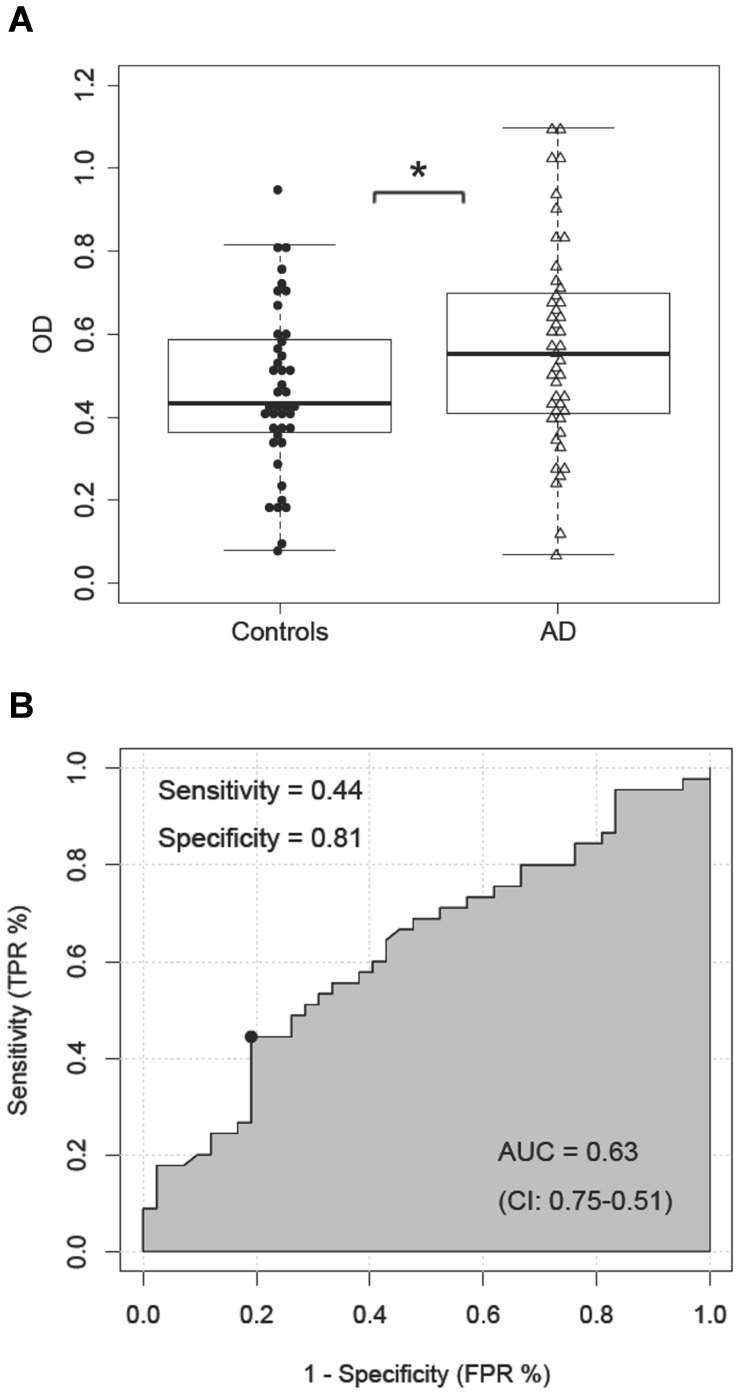
Aβ-IgG immune complexes in serum. (**A**) Comparison between the levels of Aβ-IgG immune complexes (OD at 450 nm) in serum of AD patients and control subjects; (**B**) ROC curve analysis; x-axis: 1-specificity (FPR: false positive rate), y-axis: sensitivity (TPR: true positive rate), AUC - area under the curve; * *p*≤0.05.

Higher levels of Aβ-IgG immune complexes were determined in serum of AD patients compared to the controls (*F*_(1,84)_ = 4.94, *p* = 0.03; Table 1, Figure 2A). According to ROC curve analyses, the serum Aβ-IgG levels discriminate the AD patients from the control subjects with 81% specificity and 44% sensitivity (*AUC* = 0.63, *95% CI:* 0.75–0.51; Figure 2B). When the assay sensitivity was set to 80%, specificity reached a maximum of 33%.

**Figure 3:**
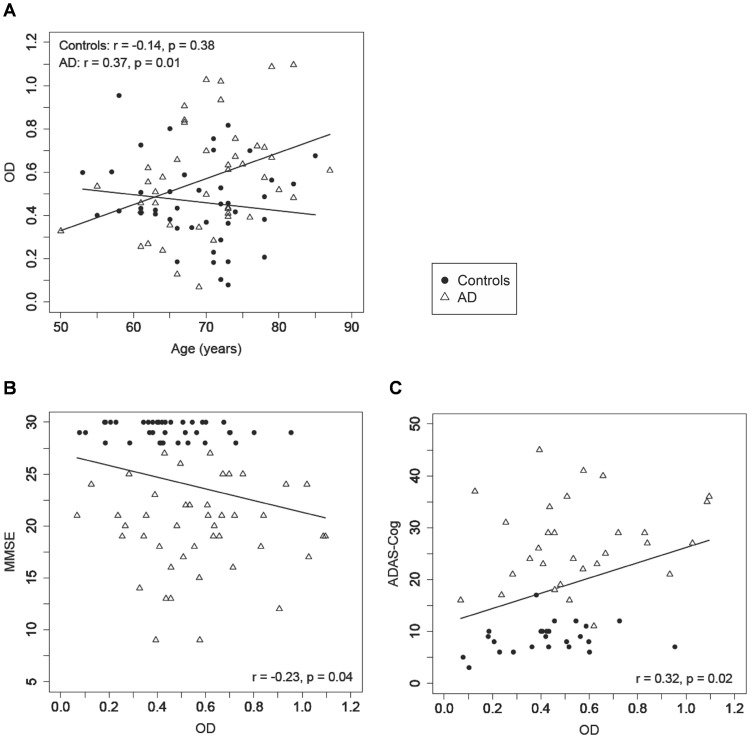
Correlations of serum levels of Aβ-IgG immune complexes with age and cognitive performance. Correlation analysis of serum levels of Aβ-IgG immune complexes (OD at 450 nm) with (**A**) age of AD patients and control subjects, respectively; (**B**) MMSE score (range 0–30 points) across all subjects and (**C**) ADAS-Cog score (range 0–70 errors) across all subjects.

The serum levels of Aβ-IgG immune complexes increased with advancing age in the AD patients (*r* = 0.37, *p* = 0.01) but not in the controls (*r* = −0.14, *p* = 0.38; Figure 3A). Furthermore, they were negatively correlated with the MMSE scores (*r* = −0.23, *p* = 0.04; Figure 3B) and positively with the ADAS-Cog scores across groups (*r* = 0.32, *p* = 0.02; Figure 3C), i.e., reaching higher values with decreasing cognitive test performance. There was no difference between the serum levels of Aβ-IgG immune complexes in the case of APOE ε4 (homo- and heterozygotes) and non-APOE ε4 carriers, either in the AD or the control group.

### Aβ-IgG Immune Complexes in CSF from AD Patients and Control Subjects

**Figure 4:**
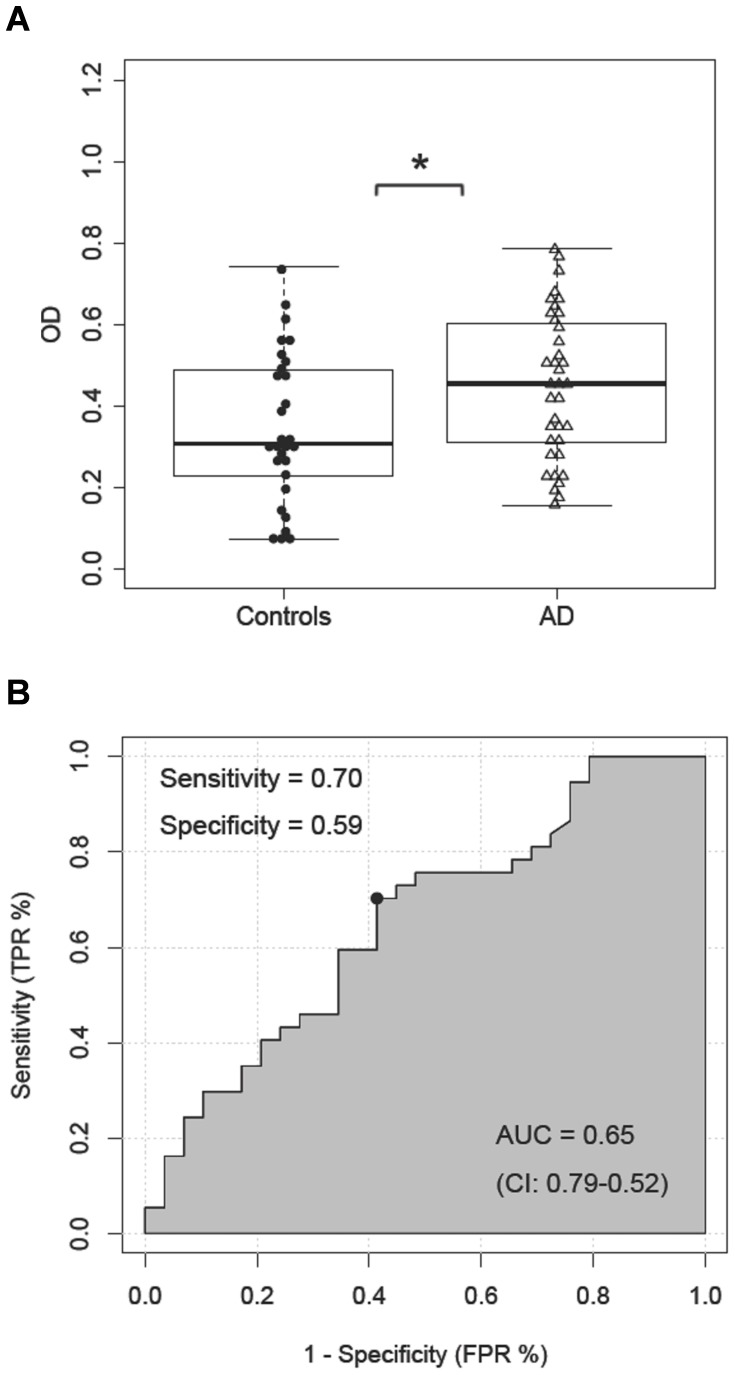
Aβ-IgG immune complexes in CSF. (**A**) Comparison between the levels of Aβ-IgG immune complexes (OD at 450 nm) in CSF of AD patients and control subjects; (**B**) ROC curve analysis; x-axis: 1-specificity (FPR: false positive rate), y-axis: sensitivity (TPR: true positive rate), AUC - area under the curve; * *p*≤0.05.

The levels of Aβ-IgG immune complexes were higher in AD patients compared to the controls (*F*_(1,63)_ = 4.98, *p* = 0.03; Table 1, Figure 4A). ROC curve analyses indicated 59% specificity and 70% sensitivity (*AUC* = 0.65, *95% CI:* 0.79–0.52; Figure 4B) for the diagnostic discrimination of the assay between AD cases and controls. When specificity of the Aβ-IgG determinations was set to 80%, sensitivity reached a maximum of 33%. When sensitivity was set to 80%, specificity reached a maximum of 31%. The ratio of the CSF to serum levels of the Aβ-IgG immune complexes showed 82% specificity and 50% sensitivity in ROC curve analysis (*AUC* = 0.67, *95% CI:* 0.83–0.50). When sensitivity was set to 80%, specificity was only 35%. The ROC curve analysis of the CSF T-tau/Aβ42 concentration ratio showed 91% specificity and 93% sensitivity (*AUC* = 0.97, *95% CI:* 0.10–0.94).

**Figure 5:**
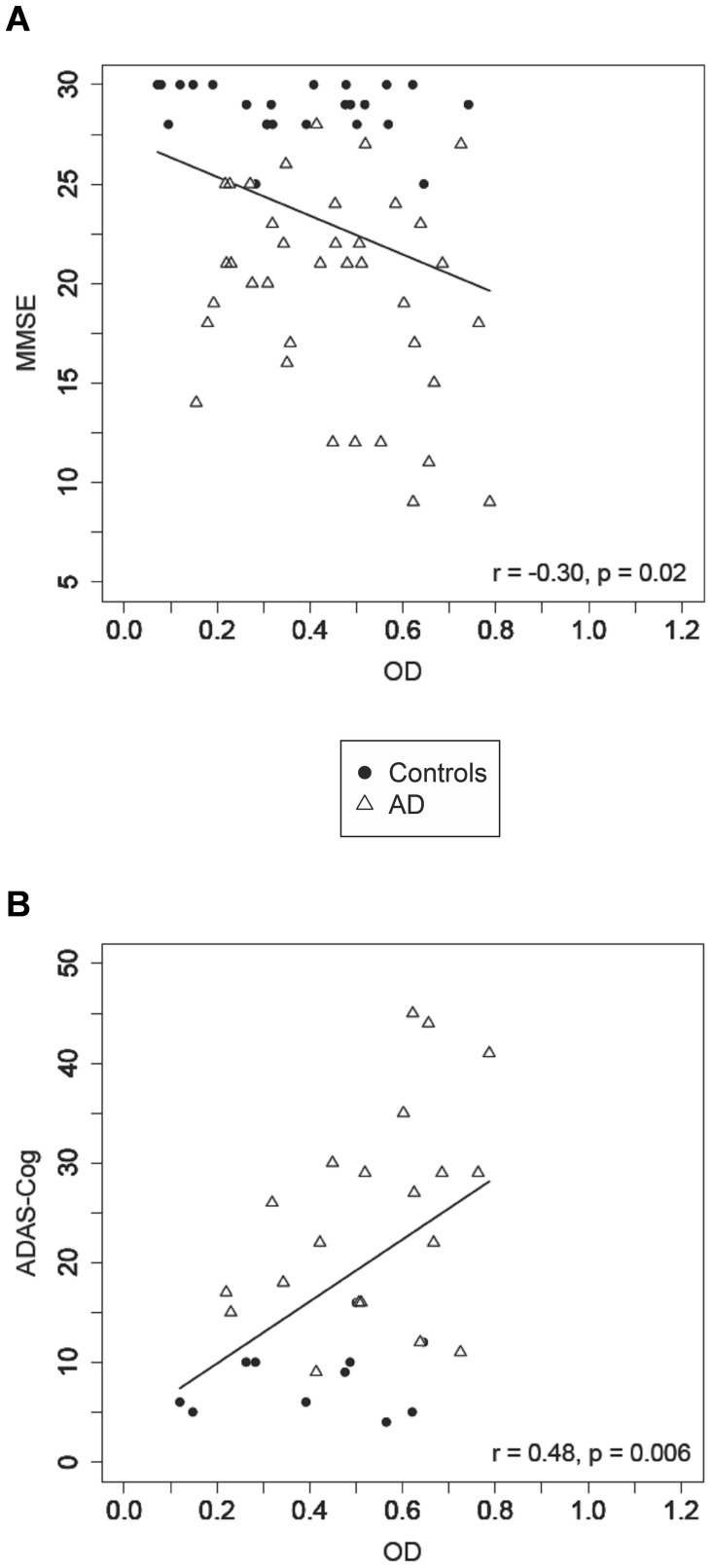
Correlations of CSF levels of Aβ-IgG immune complexes with the cognitive performance. Correlation analysis of CSF levels of Aβ-IgG immune complexes (OD at 450 nm) with (**A**) MMSE score (range 0–30 points) across all subjects and (**B**) ADAS-Cog score (range 0–70 errors) across all subjects.

The CSF levels of Aβ-IgG immune complexes across all subjects were negatively correlated with the MMSE scores (*r* = −0.30, *p* = 0.02) and positively correlated with the ADAS-Cog test scores (*r* = 0.48, *p* = 0.006), increasing with the decline of cognitive performance (Figure 5A, B). Furthermore, they were positively correlated with the Aβ42 concentration in CSF of AD patients (*r* = 0.35, *p* = 0.04; Figure S1 in the Supporting Information), but not of control subjects. A highly significant positive correlation was observed across groups between the levels of Aβ-IgG immune complexes in CSF and serum (*r* = 0.54, *p* = 0.0002; Figure S2 in the Supporting Information). There was no effect of age or APOE ε4 (homo- and heterozygote) genotype on the Aβ-IgG levels in the CSF of either AD patients or control subjects.

## Discussion

AD-related pathological processes start well before the onset of clinical manifestations [2], [4], [50], [51]. Hence, the identification and evaluation of biochemical markers that enable an early diagnosis should be of substantial value. CSF levels of Aβ42 and tau protein are currently the only reliable biomarkers for the diagnosis of AD, with sufficient sensitivity and specificity [52], while efforts to establish less invasive blood-derived biomarkers have been hitherto unsuccessful. Reports on potential serum-biomarkers for AD diagnosis have provided contradictory results and it is unclear whether changes in the periphery reflect pathologies within the brain [53]–[55]. Thus, studies of Aβ42 levels in serum of AD patients showed both reduced [56], [57] and increased values [58], [59] compared to control subjects, while other reports indicated no differences between groups [60], [61]. Also, no correlation was found between the serum Aβ42 levels and the Aβ42 levels in CSF of AD patients and healthy individuals [62], [63], the accumulation of Aβ peptides in AD brain [64] or the progression of cognitive deterioration in AD [65], [66]. Furthermore, research focused on blood protein signatures recently revealed that epidermal growth factor (EGF), platelet-derived growth factor (PDG-BB) and macrophage inflammatory protein 1δ (MIP-1δ) differentiated AD from control subjects, but not from patients with other types of dementia [67].

Physiological Aβ-autoantibodies have been detected in serum and CSF [20]–[24], [27] and generated a high interest as a potential biomarker for AD, however with hitherto inconsistent results. In AD patients compared to controls, the serum levels of free, non-antigen-bound Aβ-autoantibodies were found to be reduced [28]–[30], [34], enhanced [31] or unchanged [32], [33]. Other studies reported increased levels of Aβ-autoantibodies after acidic dissociation of preformed Aβ-immune complexes in serum of AD patients [35], [36]. Nevertheless, these results could not be reproduced by Klaver et al. [37], who found no difference in the levels of dissociated Aβ-autoantibodies between AD and control subjects.

Due to the postulated imbalance between Aβ production and removal in AD [11], we have evaluated the contribution of Aβ-autoantibodies to Aβ-clearance and the diagnostic potential of Aβ-IgG immune complexes. Our method could be applied as an alternative or complementary approach to the previously reported direct ELISAs for the determination of total Aβ-autoantibodies levels. It does not require additional sample preparation steps such as acidic dissociation and may provide valuable information on possible problems related to antibody avidity and clearance of the immune complexes. Another important aspect is the subtraction of the NSB from the OD response of each sample, a procedure previously reported only in a few ELISA studies of Aβ-autoantibodies [37]. We initially optimized and employed the sandwich ELISA protocol for the analysis of serum samples from healthy adults aged 18–89 years [41]. In the present study, the experimental procedure was also optimized for CSF analysis and applied to determine the levels of Aβ-IgG immune complexes in serum and CSF samples from a total number of 112 AD patients and age- and gender-matched control subjects. Aβ-IgG immune complexes were detected in all serum and CSF samples, suggesting a contribution of IgG-type Aβ-autoantibodies to Aβ clearance *in vivo*. Higher Aβ-IgG levels were found in both serum and CSF of AD patients compared to controls, in agreement with two previous studies [35], [36] that revealed increased total levels of Aβ-autoantibodies in AD patients. An elevated antibody production would be expected in response to Aβ accumulation, which is either due to deficient clearance mechanisms [68], [69] or to an increased formation of Aβ peptides. The latter is mainly the case in familial AD, owing to genetic mutations of amyloid precursor protein (APP) and presenilin 1 and 2, but it can also occur in sporadic AD, where it was suggested to be partially caused by the enhanced expression and activity of APP cleaving enzyme 1 (BACE 1) [70]. The progression of the disease, despite increased Aβ-IgG levels in serum and CSF of AD patients, could indicate defective clearance mechanisms, leading to the accumulation of Aβ-IgG immune complexes in AD. In healthy individuals, antigen-bound antibodies are captured by macrophages through Fc receptor-mediated recognition and transferred to mastocytes in liver or spleen for degradation during the process of “immune adhesion”, which is regulated by antibody avidity [10], [71]. A possible explanation for the apparent clearance deficiency of Aβ-IgG immune complexes is provided by the observations of Jianping et al. [72] who found the avidity of Aβ-autoantibodies to be lower in AD patients than in healthy controls and suggested that this could impair the removal of Aβ-IgG immune complexes by macrophages.

Our results further revealed that serum and CSF levels of Aβ-IgG immune complexes were negatively correlated with the cognitive performance of the study participants. Thus, subjects with higher Aβ-IgG levels had weaker performances during MMSE screening and ADAS-Cog neuropsychological testing. The increased levels of Aβ-IgG immune complexes and their inverse correlation with the cognitive status would point to a pathological process, potentially associated with defective clearance mechanisms, as discussed above. Thereby, the reported cognitive improvements of AD patients treated with IVIg [20], [23], [27] might be partially attributed to the replacement of deficient Aβ-autoantibodies by passive immunization.

In agreement with our previous work [41], serum Aβ-IgG levels were not correlated with the age of control subjects. In the AD group, however, increased age was associated with higher levels of Aβ-IgG immune complexes in serum and might therefore represent a factor for reduced Aβ clearance in AD. A positive correlation with age was also reported by Gustaw-Rothenberg et al. [36] for the difference values between the Aβ-autoantibody levels before and after acidic dissociation of the Aβ-IgG immune complexes, which might be comparable with the levels of intact Aβ-IgG immune complexes.

As shown in Figure S1 in the Supporting Information, our results also indicated a positive correlation between the CSF levels of Aβ-IgG immune complexes and Aβ42 peptide. Furthermore, we found a strong correlation across groups between the serum and CSF levels of Aβ-IgG immune complexes. Considering the dilution factors applied in ELISA, the Aβ-IgG levels were approximately 100 fold lower in CSF than in serum, suggesting that the Aβ-autoantibodies are produced and bind to Aβ mainly in the periphery.

In summary, we report here for the first time the determination of intact Aβ-IgG immune complexes in serum and CSF of AD patients and age- and gender-matched control subjects, employing a sandwich ELISA approach. Our results showed higher serum and CSF levels of Aβ-IgG immune complexes in AD patients relative to controls; however, due to the variability within groups leading to overlapping values, the Aβ-IgG levels displayed only moderate discrimination powers in ROC analyses. A possible application of serum Aβ-IgG immune complexes for AD diagnosis in a panel of blood-derived biomarkers remains to be further tested. Nevertheless, the correlation of both serum and CSF Aβ-IgG levels with the cognitive status across groups represents a valuable characteristic and it would be interesting to assess their potential use for predicting conversion to AD or evaluating the efficacy of therapeutic interventions in AD (e.g., passive immunization with intravenous immunoglobulin preparations containing Aβ-autoantibodies).

Our findings additionally suggest an increased immune response in AD, presumably associated with deficiencies in the clearance of Aβ-IgG immune complexes. A better understanding of the mechanisms causing the apparent accumulation of Aβ-IgG immune complexes could reveal in future studies new approaches for diagnosis or targeted treatment of AD.

## Supporting Information

Figure S1 **Correlation analysis between the levels of Aβ-IgG immune complexes (OD at 450 nm) and Aβ42 in CSF of AD patients.**
(TIF)

Figure S2 **Correlation analysis between the levels of Aβ-IgG immune complexes (OD at 450 nm) in serum and CSF across all subjects.**
(TIF)

Table S1 **Demographic and clinical data of Alzheimer’s disease patients (AD) and control subjects (C).**
(XLS)
