# Supplementary figures and images for: Increased Levels of Antigen-Bound β-Amyloid Autoantibodies in Serum and Cerebrospinal Fluid of Alzheimer’s Disease Patients
Source: PLoS One. 2013 Jul 18;8(7):e68996. doi: 10.1371/journal.pone.0068996 (PMC3715516; doi:10.1371/journal.pone.0068996)

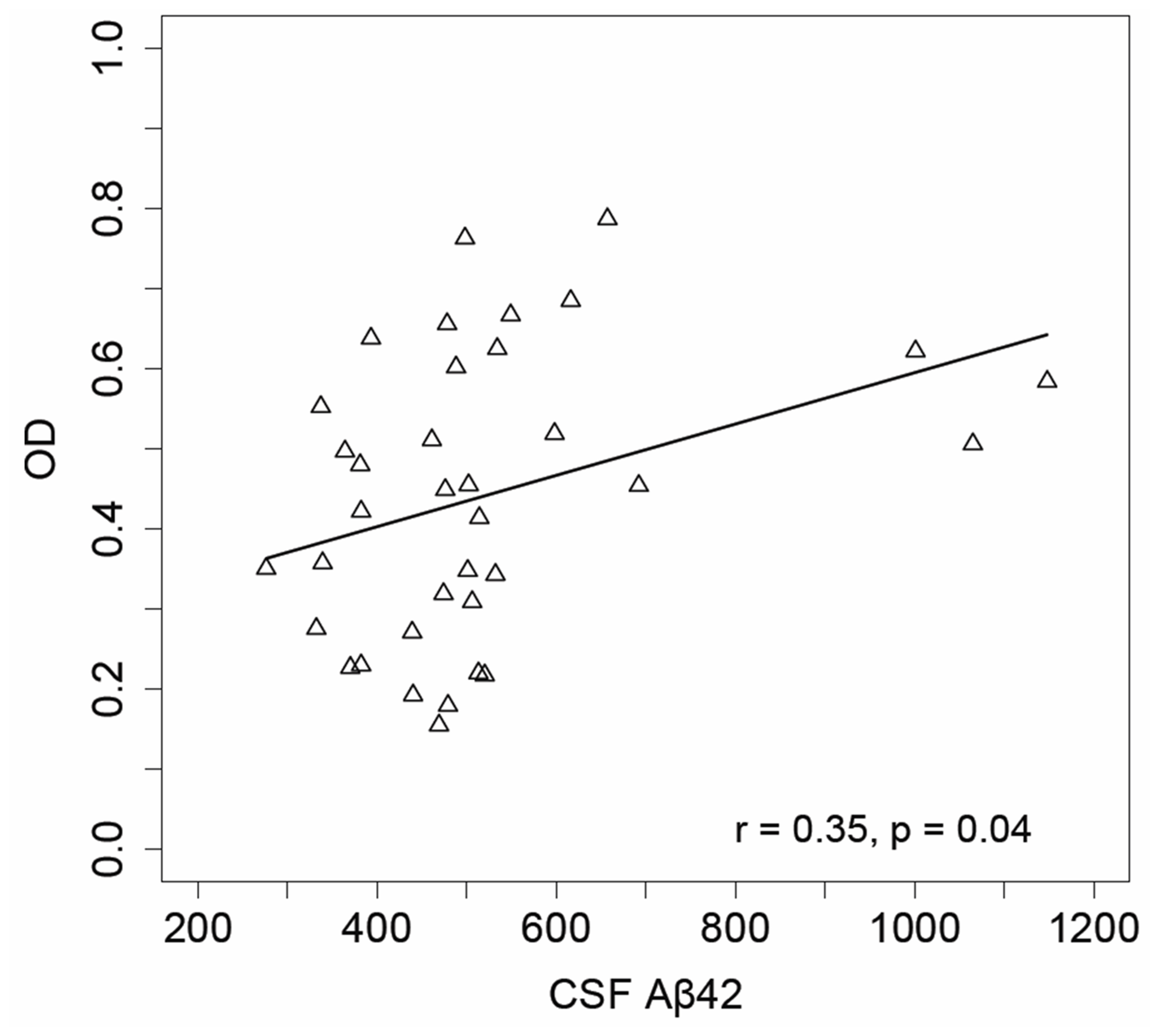

Supplement: Figure S1 — Correlation analysis between the levels of Aβ-IgG immune complexes (OD at 450 nm) and Aβ42 in CSF of AD patients. (TIF) [file pone.0068996.s001.tif]

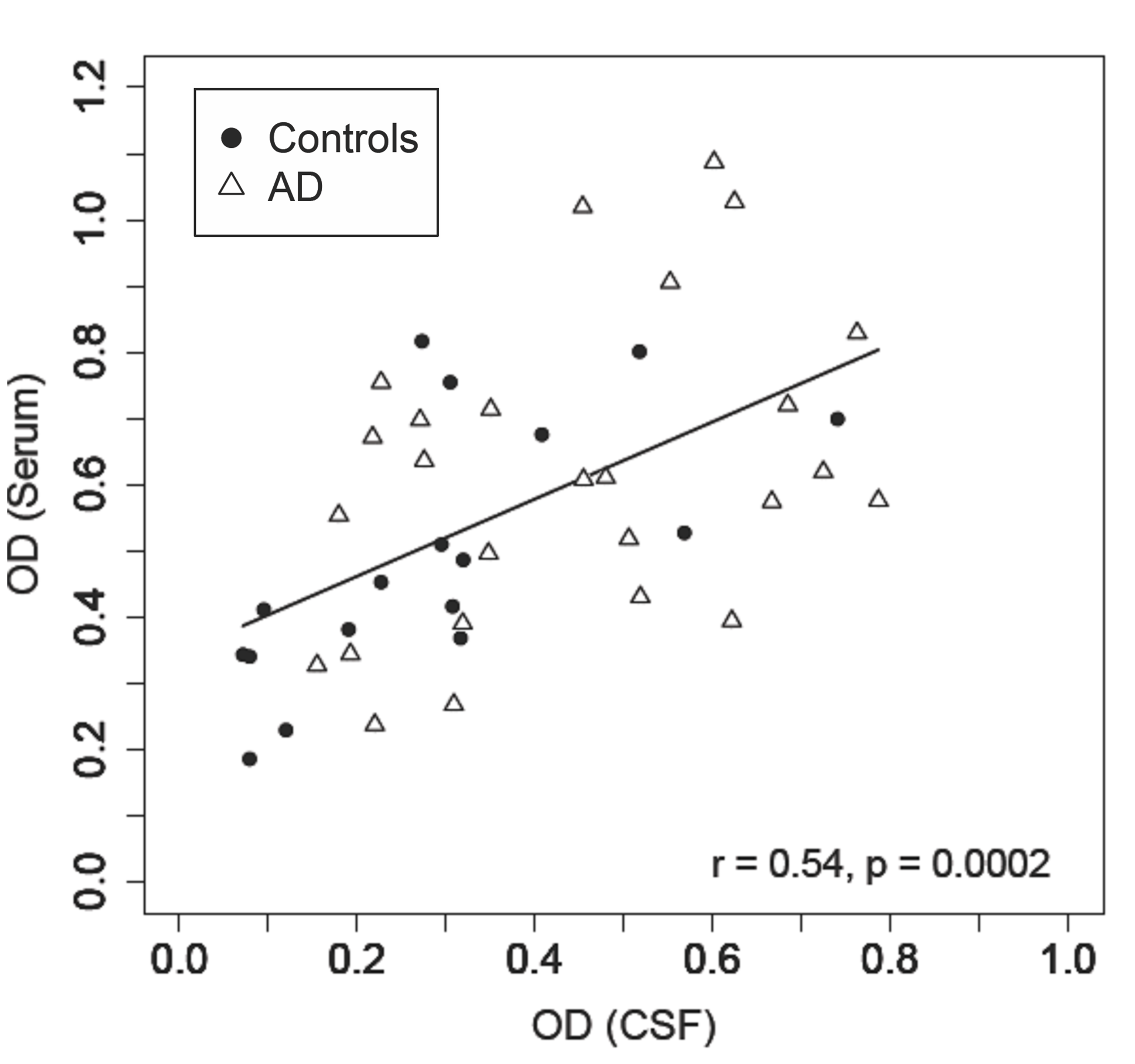

Supplement: Figure S2 — Correlation analysis between the levels of Aβ-IgG immune complexes (OD at 450 nm) in serum and CSF across all subjects. (TIF) [file pone.0068996.s002.tif]
